# Dermatomyositis following resection of thyroid and breast cancer: a case report and literature review

**DOI:** 10.3389/fonc.2025.1474481

**Published:** 2025-01-21

**Authors:** Miaomiao Yang, Jiannan Liu

**Affiliations:** Department of Oncology, Yuhuangding Hospital, Affiliated with Medical College of Qingdao University, Yantai, Shandong, China

**Keywords:** dermatomyositis, thyroid cancer, breast cancer, paraneoplastic syndrome, autoimmunity

## Abstract

**Background:**

Dermatomyositis is an idiopathic inflammatory myopathy and occurs as a paraneoplastic syndrome. Here, we report an unusual case of dermatomyositis that developed in a patient after the resection of thyroid cancer and breast cancer. The intercorrelation between dermatomyositis and neoplastic disorders was discussed.

**Case presentation:**

A 60-year-old female farmer was diagnosed with thyroid cancer and breast cancer and then developed dermatomyositis after two curative surgeries and adjuvant chemotherapy. Dermatomyositic symptoms occurred after the fifth cycle of adjuvant chemotherapy for breast cancer and deteriorated progressively. Case history, imaging, and laboratory data were reviewed. This patient subsequently received systemic immunosuppressant therapy and thus dermatomyositis gradually resolved.

**Conclusions:**

A combination of thyroid cancer and breast cancer is thought to be very rare, especially in patients who develop dermatomyositis after thyroidectomy and mastectomy. An extraordinary case was reported in this study. Potential mechanisms for the development of dermatomyositis and novel insights neoplastic autoimmune diseases were proposed.

## Introduction

Dermatomyositis is an idiopathic autoimmune-based musculocutaneous disease characterized by unique skin lesions and autoantibodies (1). It has been reported that dermatomyositis are closely associated with an underlying malignancy, accompanied by a incidence of approximately 15%-30% (2, 3). Malignancy is speculated to be a causative factor in dermatomyositis, which is considered as a paraneoplastic phenomenon (4). The malignancies most associated with dermatomyositis include carcinomas of the ovary, lung, breast, gastrointestinal tract, melanoma, and non-Hodgkin’s lymphoma (5).

Thyroid and breast cancers are endocrine-related malignancies with the highest incidence in women and often occur metachronously (6). There is a clear increase in the odds of developing either thyroid or breast cancer as a secondary malignancy, suggesting a common etiology (7). Breast cancer accounts for approximately 20% of malignancies in patients with dermatomyositis, while few reported cases of dermatomyositis associated with papillary thyroid cancer have been reported (8). There are few studies on the combination of dermatomyositis and multiple primary cancers.

Malignancy may occur before, concomitantly, or after the onset of dermatomyositis. Hence, it is inferred that there are common immunological processes linking malignancy and dermatomyositis (4). Numerous previous reports of paraneoplastic dermatomyositis have shown that the disease evolves in parallel with cancer. Anticancer therapy can remiss dermatomyositis, even without a combination of immunosuppressive drugs (9). However, the development of dermatomyositis after diagnosis or treatment of malignancy has not been fully evaluated. The molecular mechanisms by which malignancy induces inflammatory myositis are unclear (10, 11). It has been verified that chemotherapy or surgery provoke autoimmune disease (12). Cases of dermatomyositis following complete surgical removal of a tumor are extremely rare, with only two cases reported in the literature to date (13, 14). Herein, we report a rare case of a patient with thyroid and breast cancers who underwent thyroidectomy and mastectomy and then developed unexpected dermatomyositis after five cycles of adjuvant chemotherapy. Afterwards, the relevant studies were reviewed.

## Case presentation

**Figure 1:**
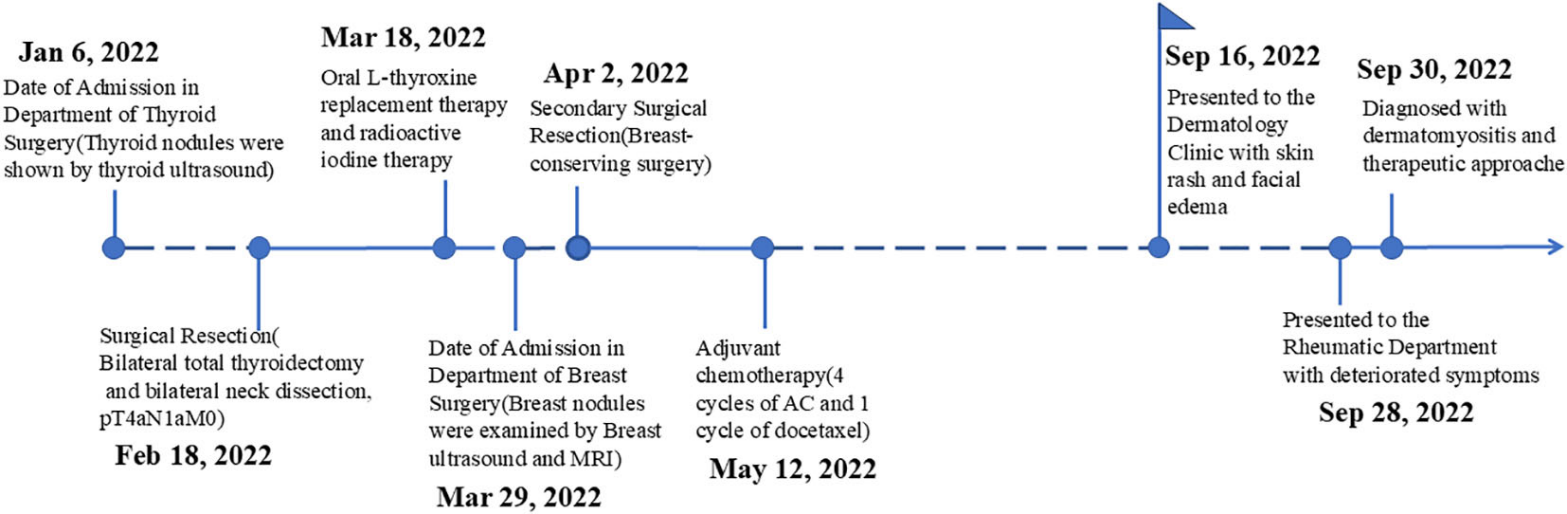
Clinical timeline for the patient.

**Figure 2:**
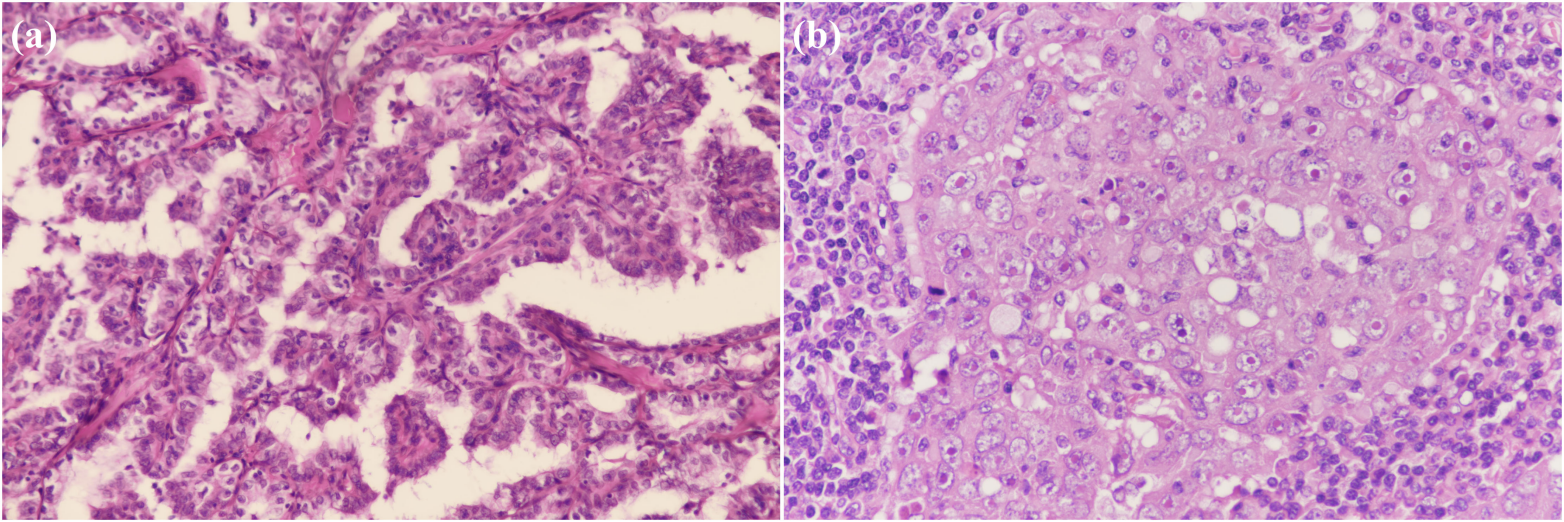
Pathological findings of the resected specimen showing, **(A)** a characteristic appearance of papillary thyroid carcinoma (PTC), with the presence of papillary structures and enlarged, irregular nuclei. (100×); **(B)** a high-grade, poorly differentiated breast invasive ductal carcinoma with irregular nuclear contours and prominent nucleoli. (200×).

On January 6, 2022, a 60-year-old female farmer with a 2-day history of dysphagia and hoarseness presented to the Yantai Yuhuangding Hospital Department of Thyroid Surgery (**Figure 1**). She had a history of shrimp and crab allergy. She was physically healthy in the past and had no history of chronic diseases such as hypertension, diabetes, cardiovascular and cerebrovascular diseases and no family hereditary disease or malignancy history. Initial examination revealed a thyroid ultrasound showing a right thyroid mass measuring 1.7×1.2 cm (TI-RADS 4b) and a left thyroid mass measuring 0.3×0.2 cm (TI-RADS 4a). Fine-needle aspiration of the thyroid mass revealed histological findings consistent with papillary thyroid carcinoma (PTC). This patient was initially diagnosed with thyroid cancer. She underwent bilateral total thyroidectomy and bilateral neck dissection on February 18, 2022, without postoperative complications. Intraoperative frozen pathology showed invasion of the laryngeal nerve and esophagus. According to the final surgical pathology (**Figure 2A**), it showed a characteristic appearance of PTC, with the presence of papillary structures and enlarged, irregular nuclei. Additionally, it was consistent with a background of lymphocytic thyroiditis, PTC in the right thyroid lobe with the largest dimension (1.5 cm), and PTC in the isthmus and left thyroid lobe with the largest dimension 0.2 cm. The tumor was staged as pT4aN1aM0. The patient received oral l-thyroxine replacement therapy. The radiation oncologist suggested proceeding with radioactive iodine therapy approximately 1 month postoperatively.

On March 29, 2022, the patient presented to our Department of Breast Surgery, and was found to have a palpable lump in the left breast that gradually increased in size during the recovery period. Breast ultrasound was carried out, showing a 1.4×0.7 cm mass with irregular polylobed contours in the left breast between 2 and 3 o’clock positions. The lesion was classified according to Breast Imaging Reporting and Data System (BI-RADS) 4b. Magnetic resonance imaging (MRI) of the breast confirmed the presence of a nodule. No distant metastases were detected, and radical correction of the left breast reservation with sentinel node biopsy was performed. The pathology report revealed Grade III invasive ductal carcinoma (**Figure 2B**). The pathological image was characterized by a high-grade, poorly differentiated tumor. The cells showed marked pleomorphism with irregular nuclear contours and prominent nucleoli. The tumor cells lacked expression of estrogen receptors (ER), progesterone receptors (PR), and human epidermal growth factor receptor 2 (HER2), which is consistent with the triple-negative phenotype. Ki-67 labeling was 90%. The tumor was classified as pT1cN0M0, according to the classification system of the American Joint Committee on Cancer, seventh edition. The patient was recommended to receive sequential administration of four cycles of docetaxel (100 mg/m^2^ every 3 weeks), followed by four cycles of doxorubicin and cyclophosphamide (AC; 60/600 mg/m^2^ every 3 weeks) as adjuvant chemotherapy. No radiation therapy, endocrine or targeted therapy were administered. Finally, she was treated with four cycles of doxorubicin plus cyclophosphamide, followed by one cycle of docetaxel. The patient tolerated the overall treatment well, with a performance status of 1. Before the sixth cycle of chemotherapy, the patient presented to the Dermatology Clinic with a 1-week history of skin rash and facial edema. The patient suffered progressive severe facial and mandibular edema and erythematous eruptions involving the face, chest, back, and upper limbs. In retrospect, she had eaten a crab 1 week previously. Given this situation, this phenomenon might be a side effect of the chemotherapy or an allergic reaction. At that time, she received loratadine, levocetirizine, and topical steroids, while the symptoms improved only briefly and slightly.

**Figure 3:**
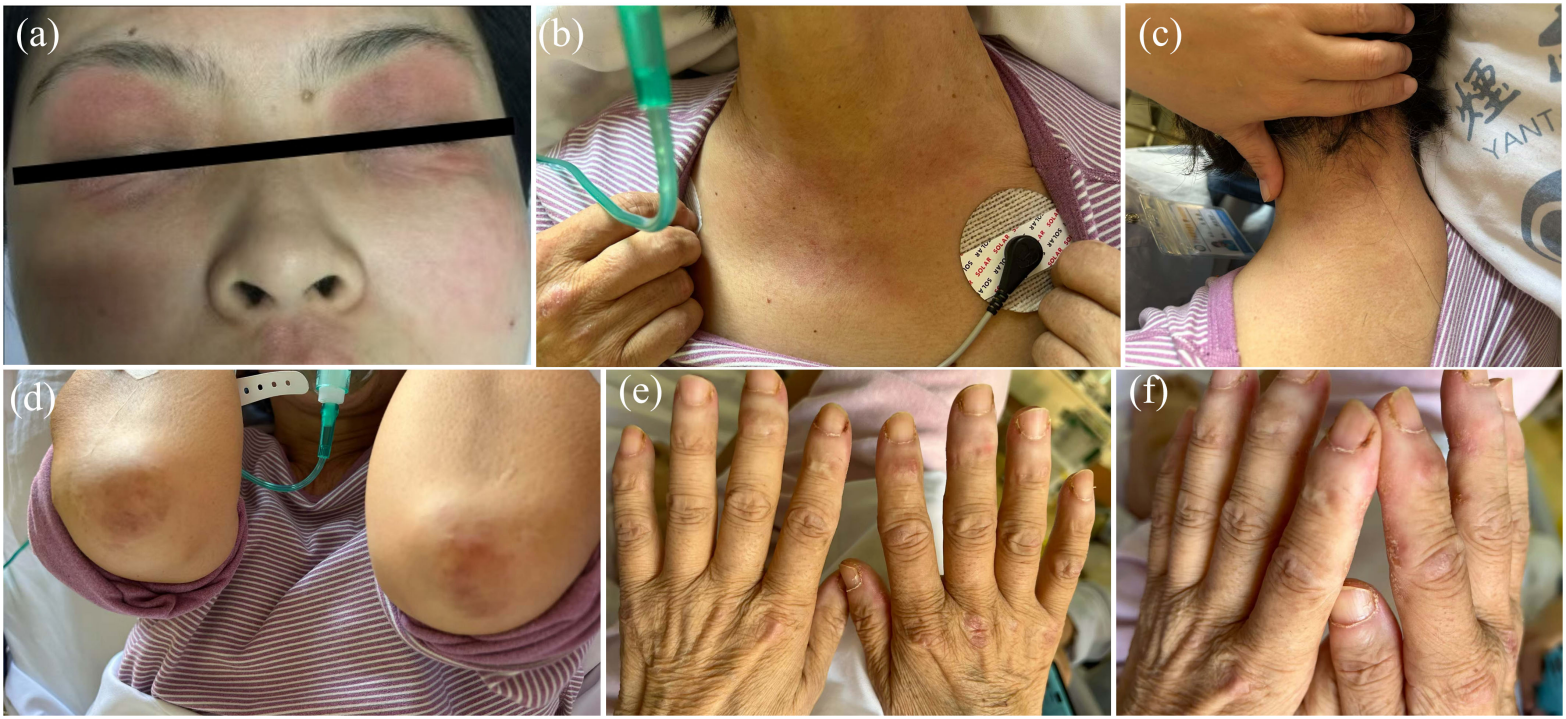
Clinical features of the patient. **(A)** Edema with heliotrope rash of the bilateral eyelids. **(B)** V-neck signs of the chest. **(C)** shawl-sign rash (macular exanthema on the back site of neck). **(D)** Gottron’s signs of the elbows. **(E)** Gottron’s signs of the dorsal side of finger joints. **(F)** Mechanic’s Hands with periungual hemorrhage and thickening of the nail cuticle.

After one week, the patient’s general condition deteriorated. She presented with progressive pruritic erythema, worsening weakness, and proximal upper- and lower-limb myalgia. She had difficulty in raising her arms, squatting up and down, and flexing her neck. Subsequently, she experienced dysphagia, cough, and hoarseness, which prompted admission to the hospital for further management. On September 28, 2022, this patient presented to the Rheumatic Department. Physical examination revealed facial edema and generalized skin eruption, heliotrope rash of the bilateral eyelids, V-neck signs of the chest, Gottron’s signs of the knees and elbows, Holster’s sign, and shawl’s sign (**Figure 3**). Muscle strength of the proximal muscles of the limbs was diminished (4/5), while the other distal limb muscle groups had normal muscle strength. The rest of the physical examination results were unremarkable.

**Figure 4:**
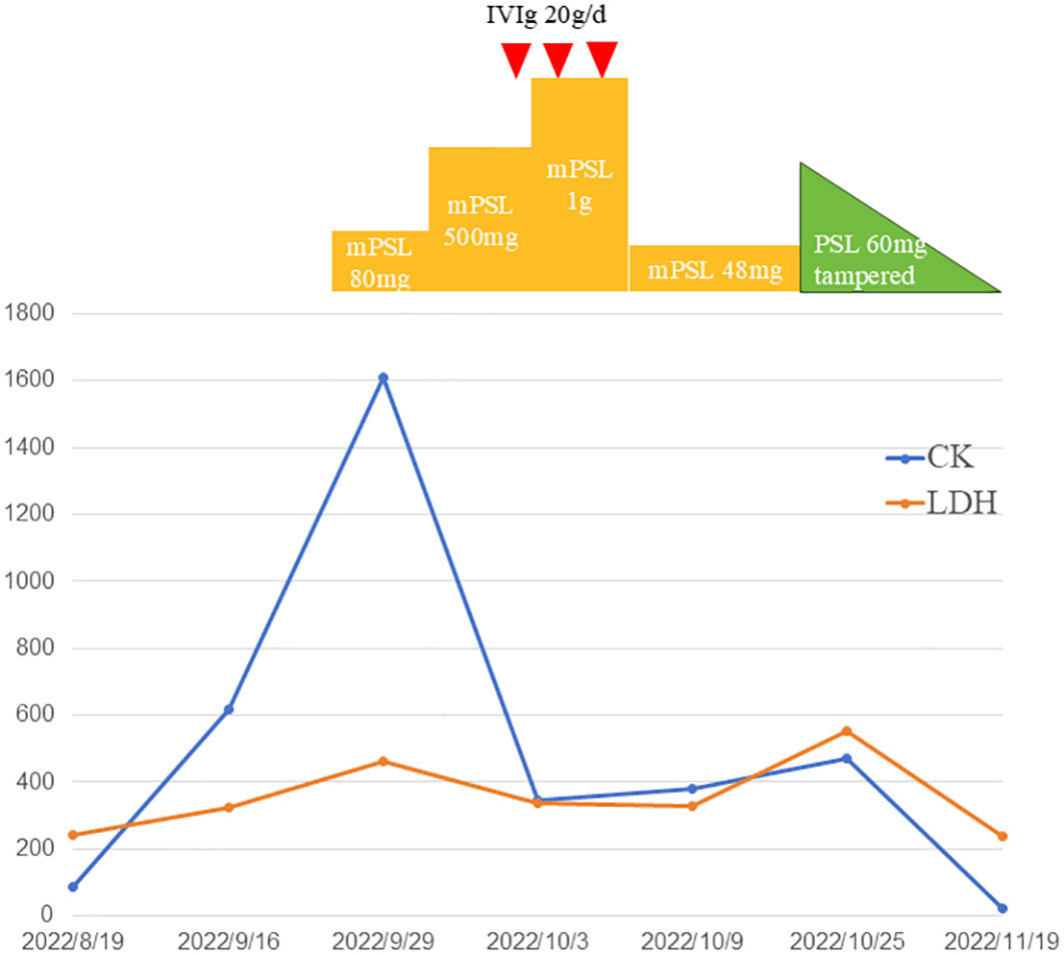
Temporal changes of creatinine kinase (CK) and lactate dehydrogenase (LDH). Treatments performed are indicated above the graph. PSL indicates prednisolone; mPSL, methylprednisolone; IVIg, intravenous immunoglobulin.

**Figure 5:**
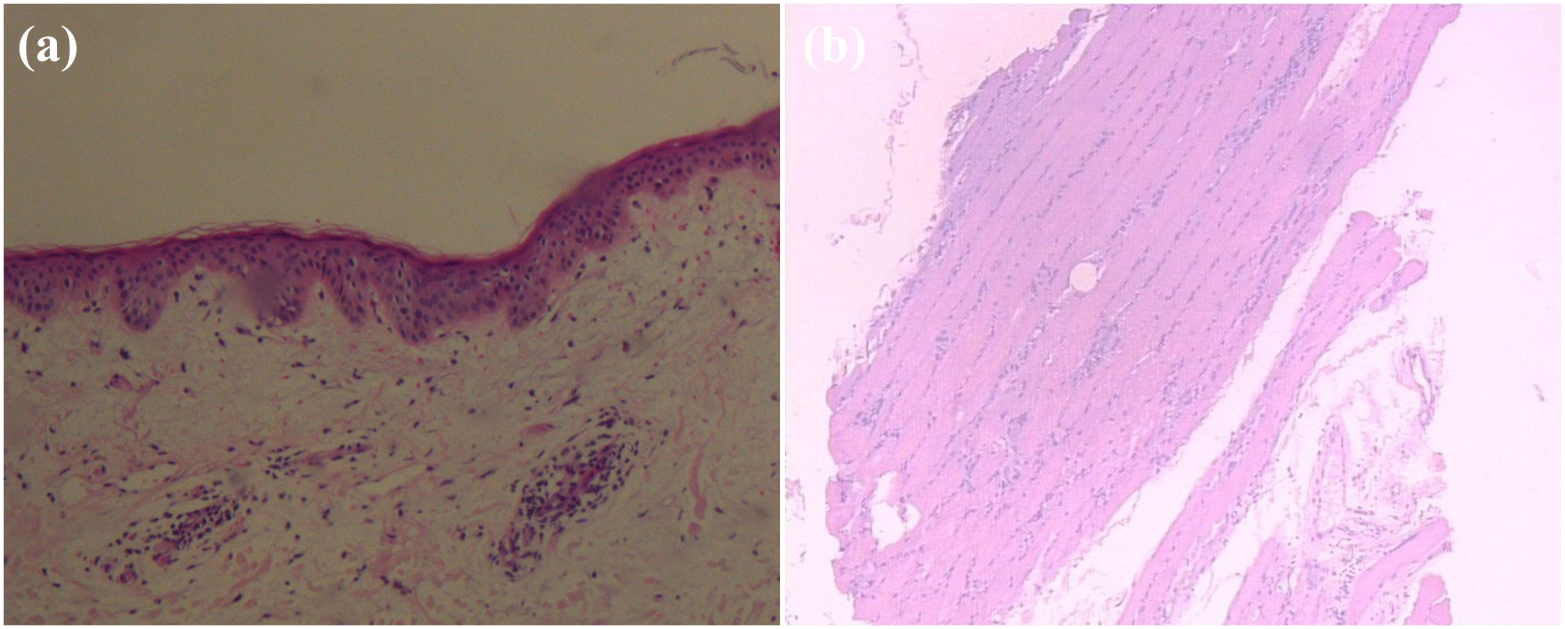
Pathological findings of the biopsy specimen showing, **(A)** cervical skin with chronic inflammatory cell infiltration around small blood vessels; **(B)** deltoid muscle biopsy with focal infiltration of chronic inflammatory cells between muscle bundles. (hematoxylin and eosin section, 100×).

Laboratory examination showed a significantly increased creatine phosphokinase (CK) level (1608 IU/L with a normal laboratory range of 40-200 IU/L, **Figure 4**), and testing for myositis−specific and myositis−associated autoantibodies showed that the antinuclear antibody was reactive (titer 1:100) with a speckled pattern, anti-Ro-52 antibody with a strong positive reaction, and anti-TIF-1γ antibody with a weak positive reaction. Other autoantibody tests including anti-u1RNP、anti-Sm、anti-SSA、anti-SSB、anti-J0-1、anti-Scl70 and anti-dsDNA were negative. The patient was scheduled for regular follow-up. CT tests of the chest and abdomen, brain MRI, and breast ultrasonography did not show any recurrence or metastasis. Cervical skin biopsy showed chronic inflammatory cell infiltration around the small blood vessels (**Figure 5A**). Deltoid muscle biopsy showed focal infiltration of chronic inflammatory cells between the muscle bundles (**Figure 5B**). Based on the clinical and histopathological examinations, the patient was diagnosed with dermatomyositis.

**Figure 6:**
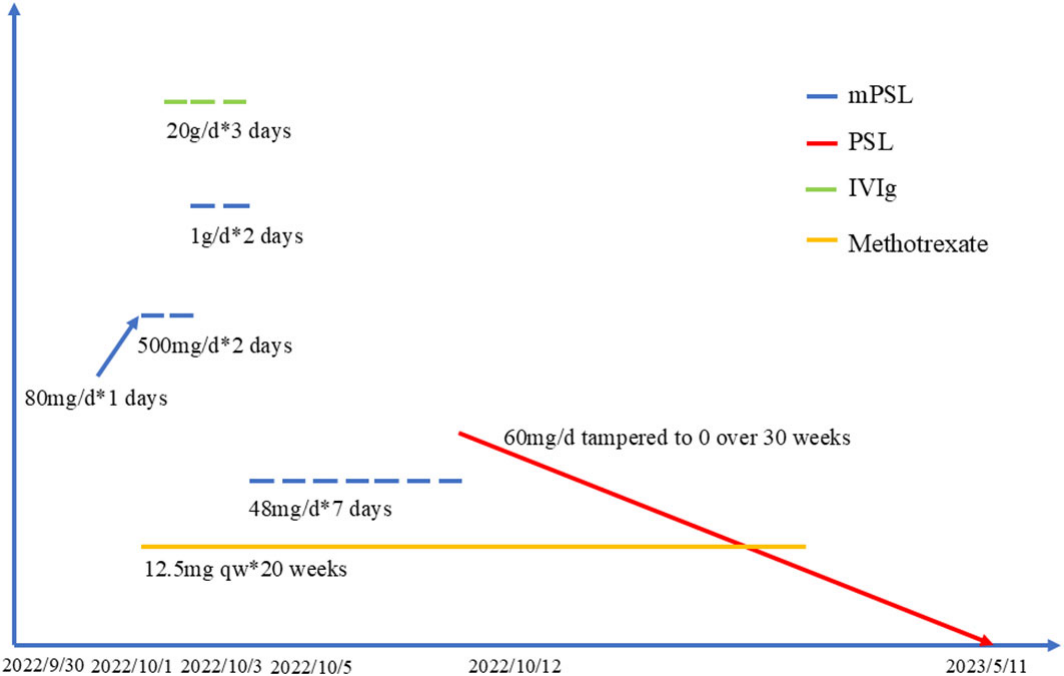
Timeline history of drug administration.

Methylprednisolone (mPSL) was administered intravenously at a dose of 80 mg/d (**Figure 6**). On the second day of the daily regimen, dysphagia and weakness developed and worsened. She had difficulty eating and drinking, and had to be supplemented with a feeding tube. The dosage of methylprednisolone was switched to 500 mg/d for 2 days and then increased to 1 g/d for 2 days. Intravenous immunoglobulin (IVIg) therapy was administered at 20 g/d for 3 days. We also used a combination of methotrexate 12.5mg/week as the immunosuppressive agent. After impulse therapy, the mPSL dosage was tapered to 48 mg/d and continued for 1 week. Her clinical symptoms and biochemical abnormalities gradually improved. After discharge from the hospital, prednisone (PSL) was initiated at a dose of 60 mg orally daily and a tapering schedule over 30 weeks, with no relapse of symptoms. Encouraging progress in disease healing was achieved. The erythematous rashes almost completely disappeared. The symptoms of weakness and dysphagia improved obviously, and the patient could eat without a nutritional tube. Her dermatomyositis symptoms did not flare up, and the remission continued. The serum CK levels also decreased to the normal range. Adjuvant chemotherapy was not continued because of a dermatomyositis episode. The patient remained in good condition, without dermatomyositis symptoms, local relapse of cancer, or development of other cancers.

## Discussion

Dermatomyositis is an idiopathic inflammatory myopathy characterized by classic cutaneous manifestations, including Gottron papules, heliotrope rash, and shawl sign (1). A series of investigations have shown that dermatomyositis is closely associated with various malignancies, such as ovary, lung, and breast being the most common tumors (5). The development of dermatomyositis may precede, coincide with, or postdate the onset of cancer. Their relationship can be directed in both ways: both diseases may contribute to the development of the other (4). Dermatomyositis with malignancy has been reported to be related to poor prognosis. The 5-year survival rate in dermatomyositis patients with cancer is significantly lower than that in overall dermatomyositis patients by 10-56% (15, 16).

As reported in a previous study, most patients with dermatomyositis suffered from one type of cancer; in a relatively small fraction of cases, multiple primary malignancies were detected (17). The occurrence of multiple primary malignancies and dermatomyositis in the same patient was unusual. Thyroid and breast cancers account for a large proportion of endocrine-related malignancies. There is an association between thyroid cancer and breast cancer, and breast cancer or thyroid cancer predisposes an individual to develop the other (6, 7). Explanations for these associations include detection bias, shared hormonal risk factors, treatment effects, and genetic susceptibility. A meta-analysis of 18 studies showed an increased risk of thyroid cancer as a secondary malignancy following breast cancer (and vice versa) compared with the background risk of other second primary malignancies (18).

There are five common dermatomyositis-specific autoantibodies: anti-Mi2, anti-TIF1-γ, anti-NXP2, anti-MDA5, and anti-SAE (19). Increased anti-TIF1-γ or anti-NXP2 antibodies can help predict the risk of cancer and have been confirmed to be a marker for paraneoplastic dermatomyositis. Anti-TIF1-γ antibodies have been detected in 50-100% of patients with cancer and dermatomyositis. According to Fujimoto et al., dermatomyositis patients with TIF-1-γ and TIF-1-α autoantibodies have a higher rate of malignancy than dermatomyositis patients with TIF-1-γ alone (11). Anti-TIF1-γ is associated with a 9.37-fold higher risk of cancer and has the strongest correlation with malignant diseases (19). In accordance with a previous study including 312 adult dermatomyositis patients, the pooled sensitivity and specificity of anti-TIF1-γ antibodies to diagnose paraneoplastic dermatomyositis was 78% and 89%, respectively. A Japanese cohort study of adult myositis suggested a possible association between anti-NXP2 antibodies and malignancy, as 37.5% of the anti-NXP2 antibody-positive patients had malignancy (20). However, no autoimmune antibody markers with high sensitivity and specificity have been certified for diagnosis. In our case, the patient had elevated CK, a positive ANA titer, and anti-TIF1-γ antibody, providing clues to possible paraneoplastic dermatomyositis.

There is no standardized treatment for cancer associated with dermatomyositis, and individualized treatment needs to be developed based on patient’s condition. Glucocorticoids are management mainstay, and intravenous immunoglobulins are used in severe cases (21). In many neoplastic dermatomyositis cases, the clinical course of the disease is similar to that of an underlying malignancy (22). Many studies have reported that symptoms related to dermatomyositis either regress or disappear completely by resection of the associated tumor (9, 14, 23).

However, our patient presented an unusual presentation of dermatomyositis. A combination of multiple primary malignancies and dermatomyositis in the same patient is thought to be extremely rare. Interestingly, this patient presented with muscular and cutaneous manifestations of dermatomyositis after radical surgical treatment of two different primary malignancies and underwent treatment with adjuvant chemotherapy. Dermatomyositis was not diagnosed immediately. The patient’s initial presentation raised the possibility of an allergic reaction, which might act as a trigger for dermatomyositis. Subsequent adjuvant chemotherapy played a role in immunosuppression and covered the potential autoimmune process in dermatomyositis. Her symptoms disappeared with a combination of steroids, IVIg, and immunosuppressive agents. Herein, we discuss the correlation between multiple primary cancers and dermatomyositis.

The mechanism of dermatomyositis involves complement deposition in the muscle microvasculature and antibody-mediated capillary destruction (24). However, the pathogenesis of dermatomyositis in malignancy has not been fully understood. Both malignancy and dermatomyositis are closely associated with immune system impairments (3). Several hypotheses regarding the concurrent occurrence of cancer and dermatomyositis were proposed, including a) autoimmunity by neoplastic antigens; b) substance secretion by tumor cells has a direct toxic effect on the muscle fibers; c) autoimmune disorder and impaired immune surveillance due to dermatomyositis; d) cross-reactive mechanisms affecting both the tumor cells and muscle fibers; and e) viral reactivation or other causative factors activating oncogenes and facilitating an abnormal immunologic response (3, 22, 25). However, no conclusive mechanism behind the association of malignancy and dermatomyositis has been established.

It was difficult to ascertain whether our case was an autoimmune process or paraneoplastic dermatomyositis. Presumably, both coincidental and true associations existed. Theoretically, tumor removal or treatment eliminates autoantigens and suppresses autoreactivity under the threshold for the development of dermatomyositis. To explain why the unexpected onset of dermatomyositis occurred after surgery and adjuvant chemotherapy in our case, the following hypotheses were proposed.

First, tumor cells release and present novel antigens that can be recognized by the immune system. In some individuals, these antigens may trigger an autoimmune response that targets not only the tumor cells but also other tissues, such as muscle fibers, leading to dermatomyositis. Although surgical treatment of the tumor tissue eliminated most of the clones, tumor antigen remained. Once an appropriate threshold for the development of dermatomyositis was attained, autoreactivity appeared. In other words, the patient had an occult autoimmune mechanism of dermatomyositis.

Second, conversion of chemotherapy regimens may also be a factor and cannot be ignored. While chemotherapy is primarily immunosuppressive, certain drugs can paradoxically unveil underlying autoimmune conditions by exposing tumor antigens or altering immune surveillance. For instance, drugs like docetaxel have been reported to induce immunogenic cell death, thereby enhancing the presentation of tumor antigens to the immune system (26). This could potentially trigger or exacerbate autoimmune conditions such as dermatomyositis. Our case discussed highlights this possibility, where a change in chemotherapy regimen coincided with the onset of dermatomyositis symptoms.

Third, molecular pathway and proteomic analyses showed a genetic overlap between cancer and dermatomyositis (27). Genetic mechanisms shared between dermatomyositis and cancer provide an opportunity to examine the role of autoimmunity in cancer development. Previous studies have identified certain polymorphisms and haplotypes that are associated with both conditions. For example, central genes such as macrophage migration inhibitor (MIF), showed significant alterations in DM patients with cancer (28). These genetic factors could predispose individuals to a dysregulated immune response, leading to the development of paraneoplastic syndromes.

Fourth, drug hypersensitivity reactions have been reported to be associated with autoimmune responses. Hypersensitivity reactions can lead to a heightened immune state, characterized by the release of various cytokines and inflammatory mediators. In a patient with an underlying predisposition to autoimmunity, such immune activation could precipitate conditions like dermatomyositis. In our case, dermatomyositis was not diagnosed immediately. The patient received a fifth cycle of chemotherapy, when it was switched from anthracycline combined with cyclophosphamide to docetaxel. During chemotherapy, the patient’s presentation initially raised the possibility of an allergic reaction, which might act as a trigger for dermatomyositis. The initial misdiagnosis of an allergic reaction in our case underscores the need for heightened clinical vigilance and comprehensive diagnostic evaluations.

Fifth, as previous studies have shown, tumor resection may cause a subsequent imbalance of Th1/Th2 immunity and induce dermatomyositis through robust inflammation (29). As a result, the concept of immune dysregulation post-tumor resection deserves further exploration. Tumor removal can lead to a sudden shift in the immune environment, potentially causing an imbalance in cytokine profiles and immune cell populations. This shift could result in a pro-inflammatory state that triggers autoimmune conditions. The delay in the onset of dermatomyositis symptoms post-surgery, as seen in the discussed case, suggests a complex interplay of factors rather than a direct cause-effect relationship with surgical stress alone.

## Conclusion

Dermatomyositis may not improve with tumor removal or treatment. It is a rare phenomenon for a patient to have both dermatomyositis and multiple primary cancers. Our case reinforces the importance of maintaining a high suspicion for the development of dermatomyositis in a patient with a history of cancer, even in the setting of radical resection and adjuvant chemotherapy. The concurrent occurrence of dermatomyositis and malignancy is likely multifactorial, involving tumor antigenicity, chemotherapeutic effects, genetic predispositions, immune dysregulation, and hypersensitivity reactions. Future research should focus on elucidating these mechanisms through advanced molecular and genetic studies. Clinicians should adopt a multidisciplinary approach, incorporating oncological, rheumatological, and immunological expertise, to optimize patient outcomes. Personalized treatment strategies, considering the unique clinical and genetic profile of each patient, are essential in managing this complex interplay between dermatomyositis and malignancy.

## Data Availability

The original contributions presented in the study are included in the article/supplementary material. Further inquiries can be directed to the corresponding authors.
